# Hyperventilation with Maintenance of Isocapnia. An “Old New” Method in Carbon Monoxide Intoxication

**DOI:** 10.1371/journal.pone.0170621

**Published:** 2017-01-20

**Authors:** Jacek Sein Anand, Daria Schetz, Wojciech Waldman, Marek Wiśniewski

**Affiliations:** 1 Department of Clinical Toxicology, Medical University of Gdańsk, Gdańsk, Poland; 2 Pomeranian Centre of Toxicology, Gdańsk, Poland; Charité - Universitätsmedizin Berlin, GERMANY

## Abstract

**Introduction:**

Exposure to carbon monoxide (CO) is among the most common causes of acute and chronic poisonings worldwide. The crucial point of treatment of such acute poisonings is to eliminate CO from the body as fast as possible. There are currently two approaches to the management of the CO intoxication: hyperbaric oxygen therapy (HOT) and normobaric oxygen therapy (NOT). HOT is highly effective and capable of achieving the CO elimination half-time (T½) as low as 15 minutes. Unfortunately this method is expensive and not always readily available. The elimination of CO with the use of NOT (T½~70 min) is slower, but treatment can be started even on the site of the exposure and continued while the patient is transported to a hospital. The aim of the study was to evaluate the effectiveness of a method using therapeutic hyperventilation with maintenance of isocapnia (IH) in the elimination of CO in volunteers exposed to CO and to compare selected gasometric and respiratory parameters during IH with the values obtained during hyperventilation with pure oxygen (“non-isocapnic hyperventilation”–NIH).

**Material and methods:**

The study involved 13 healthy, chronically-smoking volunteers. Each of them participated in two independent hyperventilation tests: IH and NIH. The levels of carboxyhemoglobin (COHb) and selected gasometric, cardiac and respiratory parameters were measured at 0, 10 and 20 minutes during both tests. Among 13 volunteers (8 women and 5 men) the initial COHb level was 5.0±1.5% (mean±SD) before the IH tests and 5.1±1.9% before the NIH tests (p>0.05). After 20 minutes of the procedures the mean COHb level was 2.9±0.9% for IH and 3.6±1.2% for NIH (p<0.01). The T½ of COHb was 29.6±12.2 min and 47.3±19.2 min respectively (p<0.01). After 10 minutes of NIH respiratory alkalosis was noted in 11 participants (84.6%). Such problem was not seen during the IH procedures. No serious adverse effects were recorded during either IH or NIH. Mild symptoms such as: dyspnea, headache and paresthesias were reported by 6 volunteers (46%) during both IH and NIH tests. It is worth noting that paresthesias were only reported during NIH, by 2 participants (15.4%).

**Conclusions:**

The elimination T½ of CO during IH was comparable with the values reported during HOT, and lower than can be achieved with NOT or NIH. No serious adverse effects were reported during IH procedures. Further studies, especially direct comparisons with NOT and HOT, are necessary to evaluate the effectiveness of IH in the treatment of acute CO poisoning.

## Introduction

Carbon monoxide (CO) is an odorless and colorless gas, which is produced from the incomplete oxidation of carbon-containing compounds. The formation of CO occurs in case of an incomplete burning of carbon-based fuels, when there is no sufficient oxygen to produce carbon dioxide (CO2). According to statistical data, CO exposure is one of the most common causes of acute poisonings in the USA. It is estimated that every year the intoxications with CO cause more than 20 000 emergency room visits [1,2]. Given the scale of this phenomenon, the US National Health Promotion and Disease Prevention Initiative “Healthy People 2010”, has listed the CO exposure surveillance as one of the major environmental health priority areas [3]. Currently there are two approaches to the management of CO poisonings: the hyperbaric oxygen therapy (HOT) and the normobaric oxygen therapy (NOT). HOT is highly effective and capable of achieving the CO elimination half-time (T½) as low as 15 minutes, but unfortunately the method is expensive and not always readily available. NOT seems to be much slower (T½~70 min), but in contrast to HOT treatment can be started even on the site of the exposure and continued while the patient is transported to a hospital [4]. Owing to the frequency of intoxications with CO, there is a need for a simple, readily available, cheap and clinically effective method for faster elimination of CO from the human body than it is possible with NOT. In the 1920s a carbon dioxide (CO2) and oxygen (O2) mixture was successfully used to treat victims of carbon monoxide poisonings. The mixture, called Carbogen, was stored in tanks, in which the O2/CO2 ratio was fixed (typically 6% CO2 and 94% O2). It was administered to spontaneously breathing victims of CO poisonings to induce hyperventilation through hypercapnia. Unfortunately the inability to regulate the CO2/O2 ratio was associated with a risk of excessive hypercapnia and potentially fatal acidosis, particularly in patients with increased lactate levels and/or impaired respiratory drive. Despite this side effect, the method was successful in many cases, but in the 1960s, with the growing popularity of HOT, Carbogen was gradually withdrawn from the medical use [5]. In 1999 researchers from Toronto General Hospital introduced a new, safer method to achieve a significant increase of ventilation while maintaining blood partial pressure of carbon dioxide (paCO2) within the normal range. They invented a pressure-operated device (ClearMate), which produces a variable CO2/O2 mixture according to the patient’s current alveolar ventilation (VA). The method is called isocapnic hyperventilation (IH) because CO2 is added to the breathing mixture only when the patient’s ventilation is increased above the basal value, so there is no risk of hypercapnia [5].The aim of our study was to evaluate the effectiveness of IH in volunteers who were exposed to CO from cigarette smoke and to compare selected gasometric and respiratory parameters with the results obtained during hyperventilation with 100% O2 (“non-isocapnic hyperventilation”–NIH).

## Materials and Methods

**Fig 1:**
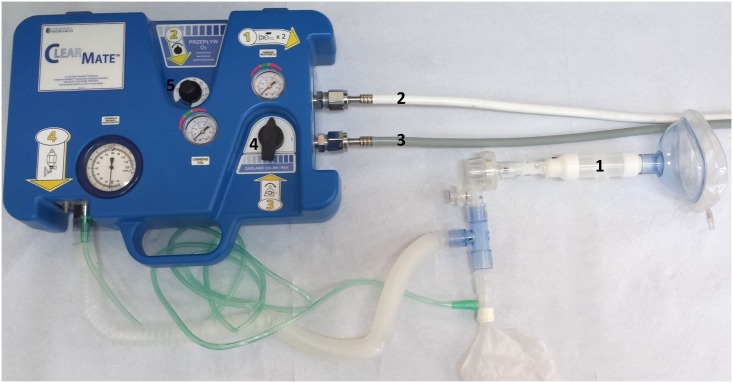
The ClearMate device and the breathing circuit used in the study. The device used during the study and the elements of the breathing circuit: 1- spirometer flow head, 2 –oxygen supply, 3 –carbon dioxide supply, 4 –carbon dioxide supply on/off switch, 5 –oxygen flow rate controller.

The study was approved by the local bioethics committee (Independent Bioethics Commission for Research at Medical University of Gdańsk, approval NKEBN/40/2012). The participants were 13 healthy, chronically smoking volunteers who gave a written informed consent to participate in the trial. ClearMate (Thornhill Research Inc., Canada), a pressure-operated device, which produces a variable CO2/O2 mixture according to the current alveolar ventilation (VA) was used in the study. The method allows to achieve a significant increase of VA while maintaining the blood partial pressure of carbon dioxide (paCO2) within the normal range. This is achieved by providing a constant 100% O2 flow, adjusted to match the metabolic CO2 production and supplying the 6% CO2 in O2 mixture by the demand regulator. The ventilation exceeding the 100% O2 flow is composed only of the CO2/O2 mixture which does not contribute to the CO2 diffusion gradient between the capillary blood and the alveoli, so that paCO2 is unchanged by any increase in ventilation [6]. It is possible to turn off the supply of CO2 to the device and then only 100% O2 is delivered both to the reservoir and through the demand valve. This ability was used in our study to compare hyperventilation with and without isocapnia (IH vs. NIH). During the study all the participants underwent two independent hyperventilation procedures: IH and NIH. Each session lasted 20 minutes. The volunteers were coached to hyperventilate until the appearance of side effects. They were informed about their current minute ventilation throughout the procedures and were instructed to increase their tidal volume and to decrease the respiratory rate when necessary. At the start of the procedures the flow of 100% O2 to the reservoir was set according to the subject’s weight (0.075 L/min/kg) and it was later adjusted manually according to a continuous capnometry measurement, to maintain the end-tidal CO2 between 45 and 50 mmHg. The initial carboxyhemoglobin (COHb) level was measured in capillary blood at least 10 minutes after the last exposure to cigarette smoke to achieve full CO distribution. Selected gasometric (pH, paCO2, paO2 in capillary blood) and cardiac parameters (heart rate, SBP, DBP) were recorded during both tests at 0, 10 and 20 minutes of the procedures. Respiratory parameters, including TV, VE, and f, were continuously registered using the START 200 M ergospirometer (MES Cracow, Poland). The spirometer flow head was incorporated into the breathing circuit between the face mask and the non-return valve. Carbon dioxide level in exhaled air was monitored continuously with an EMMA capnometer (Massimo Corp., USA) and recorded at 0, 10 and 20 minutes of the procedures. The ClearMate device and the breathing circuit used during this study are shown in Fig 1. The hyperventilation procedures were performed twice with each study subject according to the experimental protocol. During the first procedure CO2 was added to the respiratory mixture (IH) and during the second one the CO2 supply was turned off and only 100% O2 was used (NIH). The time interval between the sessions lasted 7 days for all study subjects. Because all volunteers had a background in medical sciences and the informed consent clearly outlined the potential side effects of hyperventilation (especially during NIH) we decided not to blind the procedures. We chose to perform the IH procedures before the NIH ones to minimize the effect of training on the results. We believe that if the sequence was reversed it would favor IH over NIH. We also wanted to reflect the fact that in a clinical setting of CO poisoning IH will be the patients’ first attempt at therapeutic hyperventilation. Statistical analysis was performed using the STATISTICA 10 software (StatSoft) and t-Student’s test for paired samples, t-Student’s test for independent samples and McNemar’s test were used.

## Results

**Fig 2:**
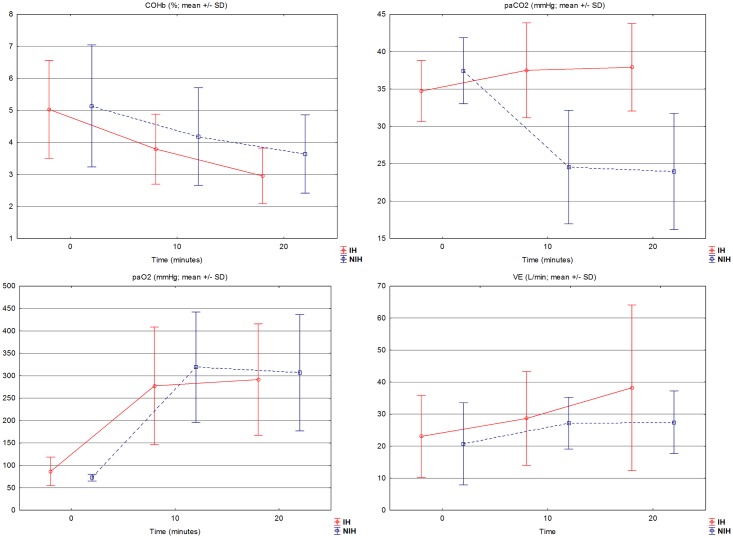
Selected parameters (COHb, paCO2, paO2 and VE) during the procedures (mean ± SD). Comparison of selected parameters during IH and NIH at 0, 10 and 20 minutes: COHb—carboxyhemoglobin level (%), paCO2 –partial pressure of carbon dioxide in capillary blood (mmHg), paO2—partial pressure of oxygen in capillary blood (mmHg), VE—total minute ventilation (L/min).

The study group included 13 volunteers aged 25–64 (mean 41.1; SD ± 9.6) years. There were 8 women aged 33–49 (39.5±5.1) years; and 5 men aged 25–64 (43.6±14.9) years. The age difference between sexes was not statistically significant (p>0.05). The average number of cigarettes smoked per day was 20.5±9 in women and 21.8±8.3 in men (p>0.05). The mean COHb level measured at the start of hyperventilation procedure was 5.0±1.5% before the IH tests and 5.1±1.9% before the NIH tests (p>0.05). After 20 minutes of the tests (final examination) the mean COHb level was 2.9±0.9% for IH and 3.6±1.2% for NIH (p<0.01). The average T½ of COHb was 29.6±12.2 min during IH and 47.3±19.2 min during NIH (p<0.01). After 10 minutes of NIH respiratory alkalosis (pH>7.45) was noted in 11 participants (84.6%) and the difference in pH of capillary blood between IH (7.41±0.06) and NIH (7.55±0.09) was statistically significant (p<0.01). Total ventilation (VE) during the entire procedure for IH and NIH was 620.2±300.8 L and 569.1±195.1 L respectively (p>0.05). Total alveolar ventilation (VA) was: 559.1±297.8 L and 483±210.6 L respectively (p>0.05). The average paCO2 after 10 minutes of IH (37.5±6.4 mm Hg) was higher compared to NIH (23±10.2 mm Hg) (p<0.01). In 6 participants (4 women and 2 men) there was no increase of ventilation during IH compared with NIH. These volunteers were significantly older than the rest of the group (46.7±9.8 vs. 36.3±7 years; p<0.05) and had a longer history of cigarette smoking (24.3±13.2 vs. 10.8±5.1 years; p<0.05). Even though no increase in ventilation was observed during IH compared to NIH this subgroup also achieved faster CO elimination during IH than during NIH. The elimination half-life of COHb was 35.2±13.1 and 60.4±18.7 respectively (p<0.05). There were no statistically significant differences in systolic and diastolic blood pressure between IH and NIH. No serious side effects necessitating interruption of the procedures were recorded. Mild symptoms such as: dyspnea, headache or paresthesias were reported by 6 volunteers (46%) during both IH and NIH tests. It is worth noting that paresthesias occurred only during NIH procedures and were reported by 2 women (15.4%). The changes of selected parameters during the procedures are presented in Fig 2.

## Discussion

In circumstances when the level of CO in inhaled air is increased a high partial pressure differential between air and blood occurs. Diffusion of CO by the lungs is a completely passive process which follows through the alveolar-capillary barrier and across the red blood cells membrane into the red blood cells stroma, where rapid and reversible binding of CO to the hemoglobin occurs. CO affinity to human hemoglobin is 218 times greater than that of O2, and upon contact with CO a stable complex of CO and hemoglobin, known as carboxyhemoglobin (COHb), is formed. When air free of CO is inhaled, the pressure differential is reversed and CO is released into the alveoli. It is worth noting, that in the case of an environmental exposure the air-blood gradient of CO is much higher than the blood-air gradient. For this reason the uptake of CO from ambient air is faster than the process of its elimination from the body. The diffusing capacity of the lung for CO can be affected by many factors such as: hemoglobin concentration, cardiac output, paCO2, COHb concentration, blood flow, body position, paO2 as well as a number of diseases, especially restrictive lung diseases and chronic obstructive pulmonary disease [4, 7–9]. For this reason all the subjects chosen for this pilot study were healthy. We decided to enroll chronic smokers because COHb level in this group may be elevated up to 8% due to the exposure to cigarette smoke. The COHb levels in the studied group at the beginning of both IH and NIH tests were respectively 5.0±1.5% and 5.1±1.9%. Carboxyhemoglobin cannot be accurately measured by a pulse oximeter, since displayed saturation level equals the sum of the oxyhemoglobin (O2Hb) and COHb [10]. In our study gasometric analysis of arterialized capillary blood sample was chosen because this procedure is less painful and harmful than arterial sampling. Moreover, according to the medical literature, this method also accurately reflects both arterial paCO2 and paO2 [11]. In our study, the initial COHb concentration in the capillary blood was measured at least 10 minutes after the last exposure to cigarette smoke This was due to the fact, that carboxyhemoglobin does not appear in blood immediately after exposure to CO. The study by Benignus et al. has shown that the COHb equilibrium was not immediate within the human blood stream. During the 5 minutes inhalation of high doses of CO by men, there was a 1–2 minute delay in the appearance of venous COHb. Although venous concentration of COHb during CO exposure was lower than arterial one, they converged 10 minutes after cessation of the CO inhalation [12]. During atmospheric oxygen breathing the mean half-life of COHb in human body is 5–6 hours. It is well known that hyperventilation increases the rate of elimination of volatile substances such as CO. The main limitation of this method is that the human body’s CO2 production is constant and arterial carbon dioxide (paCO2) is inversely proportional to alveolar ventilation (VA). During the increase in VA, there is a decrease in paCO2, which may cause cerebral vasospasm and brain ischemia. In one study, it was suggested, that maintaining isocapnia during O2 therapy in a case of CO intoxication may be crucial to decrease the frequency of neurological sequelae of the CO exposure, owing to CO2 regulatory effects on cerebral blood flow [13]. Currently the most effective method used in the case of CO intoxication is the hyperbaric oxygen therapy (HOT), which is able to achieve a reduction of half-life of COHb to 15–23 minutes [14]. However the availability of HOT is limited worldwide and most patients exposed to CO are treated with the normobaric oxygen therapy (NOT). In our study we measured the level of COHb at 0, 10 and 20 minutes of the procedures to calculate the T½ of CO elimination during IH and NIH. The half-life of COHb achieved during IH in our study was 29.6±12.2 minutes which is closer to values reported for HOT than those for NOT (T½~70 min). According to our results, we believe that the potential effectiveness of IH in the elimination of CO is comparable to HOT. During the study it was noted that some participants were unable to increase their ventilation adequately both during IH and NIH due to excessive respiratory effort. Statistical analysis showed that these participants were older and had a longer history of cigarette smoking. Even though no increase in ventilation was present in this subgroup, the enhanced elimination of CO during IH compared to NIH was also achieved.

### Limitations of the study

This is a pilot study designed to verify the feasibility of IH in spontaneously breathing patients. The ClearMate device was originally intended to be used in comatose patients, ventilated with an AMBU bag or by a mechanical ventilator. In such cases the ability to hyperventilate would depend only on the rescuers. Since most victims of CO poisoning treated in our facility are not comatose, we wanted to verify if it was possible to maintain IH over a significant amount of time during spontaneous breathing and if it would enhance the elimination of CO from the body. The study is based on the clinical data obtained from healthy volunteers who were encouraged to breathe deeply. Although the participants were coached before and during the procedures, some of them found it difficult to achieve an adequate level of hyperventilation due to the increased respiratory effort. In order to minimize the number of blood samplings, the study protocol did not include standard normobaric oxygen therapy without hyperventilation (NOT), but it is highly unlikely that CO elimination during NOT would be faster than during NIH.

## Conclusions

The elimination T½ of CO during IH was comparable with the values reported during HOT, and lower than can be achieved with NOT or NIH. No serious adverse effects were reported during IH procedures. Further studies, especially direct comparisons with NOT and HOT, are necessary to evaluate the effectiveness of IH in the treatment of acute CO poisoning.

## Supporting Information

S1 File Gasometric and spirometric data. The spreadsheet containing all the data used for analysis in this study.
(XLSX)
